# Why is DIC a Rare Diagnosis in the 21^st^ Century?

**DOI:** 10.14789/jmj.JMJ24-0005-P

**Published:** 2024-03-28

**Authors:** JECKO THACHIL, TOSHIAKI IBA, ECATERINA SCARLATESCU, JERROLD H. LEVY

**Affiliations:** 1 Department of Haematology, Manchester University Hospitals, Manchester, UK; 1 Department of Haematology, Manchester University Hospitals, Manchester, UK; 2 Department of Emergency and Disaster Medicine, Juntendo University Graduate School of Medicine, Tokyo, Japan; 2 Department of Emergency and Disaster Medicine, Juntendo University Graduate School of Medicine, Tokyo, Japan; 3 University of Medicine and Pharmacy “Carol Davila,” Bucharest, Bucharest, Romania; 3 University of Medicine and Pharmacy “Carol Davila,” Bucharest, Bucharest, Romania; 4 Department of Anaesthesia and Intensive Care, Fundeni Clinical Institute, Bucharest, Romania; 4 Department of Anaesthesia and Intensive Care, Fundeni Clinical Institute, Bucharest, Romania; 5 Department of Anesthesiology, Critical Care, and Surgery, Duke University School of Medicine, Durham, NC, USA; 5 Department of Anesthesiology, Critical Care, and Surgery, Duke University School of Medicine, Durham, NC, USA

**Keywords:** disseminated intravascular coagulation, sepsis, diagnostic criteria, bleeding

## Abstract

Disseminated Intravascular Coagulation (DIC) has been a common diagnosis made by health care givers since the dawn of the 20^th^ century. However, currently, this diagnosis is entertained rarely in clinical settings that can predispose to this complication. The incidence of four common clinical scenarios traditionally associated with DIC, sepsis, trauma, obstetrical disorders, and cancers, are on the increase due to better diagnostics and management strategies, but DIC is rarely diagnosed in these disease categories currently. The authors suggest the rarity of a DIC diagnosis is due to varied understanding of the pathophysiology of this condition. In this perspectives, we would like to present reasons for this change in consideration and encourage caregivers to consider a DIC diagnosis at an early stage based on new criteria to help patients benefit from available treatments.

## Definition of DIC

Disseminated Intravascular Coagulation (DIC) is defined by the International Society on Thrombosis and Haemostasis (ISTH) as an acquired syndrome characterized by the intravascular activation of coagulation without a specific localization and arising from different causes. It can originate from and cause damage to the microvasculature; if the damage is sufficiently severe, organ dysfunction can result ^1)^. The different subcomponents of this definition have to be satisfied for the diagnosis of DIC, and it is useful to examine them in detail.

Firstly, DIC is an acquired syndrome that arises from different causes, and as such, the diagnosis should ONLY be entertained in patients with an underlying trigger; and for the same reason, the diagnosis SHOULD be entertained when a predisposing clinical situation exists in a patient who may have clinical and laboratory evidence of dysregulated coagulation activation. Four common causes were described earlier, while other clinical situations with dysregulated coagulation activation can also lead to DIC ^2)^.

Loss of localization and intravascular coagulation activation are the other two crucial components of DIC pathogenesis. The physiological process of clot formation at the site of endothelial injury, which is always limited to the vessel wall, becomes pathological in DIC, wherein thrombus formation is not localized and starts to develop and propagate intravascularly. This “intravascular dissemination” is detected by laboratory tests which can point to uncontrolled thrombin generation and form the basis of DIC diagnostic criteria ^3)^.

The third and often overlooked component of the definition is the important role of the microvasculature. Although studied in elaborate detail by several basic science researchers, hemostasis perturbation occurring at the microvascular endothelium is not easily translatable to clinical practice ^4)^. In this context, the last part of the definition may, however, assist. The pathological process that originates from the microvasculature in DIC can result in organ dysfunction if sufficiently severe. In other words, impairment of organ (or multiorgan) function can be the “clinical” manifestation of DIC. In routine clinical practice, the onset of organ impairment is seen either clinically for acute lung injury or for neurological manifestations such as confusion- as examples or based on laboratory parameters as in the case of abnormal renal function. However, in patients who have a well-known trigger, organ dysfunction may be an early sign of DIC in the absence of clear alternate explanations. Unfortunately, a DIC diagnosis is often not entertained early in a disease course but rather later when there is multisystem thrombosis, a stage where therapeutic interventions are unlikely to be of benefit ^5)^.

## Laboratory criteria for DIC

Diagnostic criteria for DIC were developed by the ISTH two decades ago that included platelet count, prothrombin time, plasma fibrinogen, and a fibrinolytic biomarker like D-dimer ^1)^. The British Society of Haematology guidelines recommended repeating these tests since one set of tests would only provide a snapshot of the pathological process, while serial testing helps in understanding the worsening or improvement of the DIC ^6)^. One of the prominent issues in the underdiagnosis of DIC is the lack of dependence on these easily available tests in patients with likely triggers for DIC. This is evident in the huge difference in the number of patients diagnosed with DIC in Japan, where reliance on diagnostic criteria like the Japan Ministry of Health and Welfare (JMHW) criteria (developed much before the ISTH) and the Japanese Association for Acute Medicine (JAAM) criteria guide the physicians to diagnose more patients with DIC ^7)^. A recent cohort study performed in Japan reported the prevalence of DIC was 50.9% in septic patients, and the patients with DIC showed a higher incidence of multiple organ dysfunction (32.0% vs. 13.1%) and worse mortality (24.8% vs. 17.5%) ^8)^. Several other reasons may be considered for the low rate of DIC diagnosis outside Japan

• DIC is usually a complication of sepsis, trauma, cancer, or obstetrical pathologies. This means a good understanding of the DIC pathophysiology should be present among infectious disease doctors, intensive care physicians, trauma experts, oncologists, and obstetricians.

• Infrequent use of DIC diagnostic criteria by these specialists

• Attribution of abnormal laboratory results to “other” reasons. For example, thrombocytopenia is a very common presentation in critical care units and oncology patients, prolonged prothrombin time may be due to vitamin K deficiency or liver disease, increased fibrinogen and fibrinolytic biomarkers occur in many conditions requiring hospital admission, including the DIC triggers

• Entertaining the diagnosis of DIC only when the patient has multi-system thrombosis or uncontrollable bleeding when therapeutic measures are futile.

**Figure 1:**
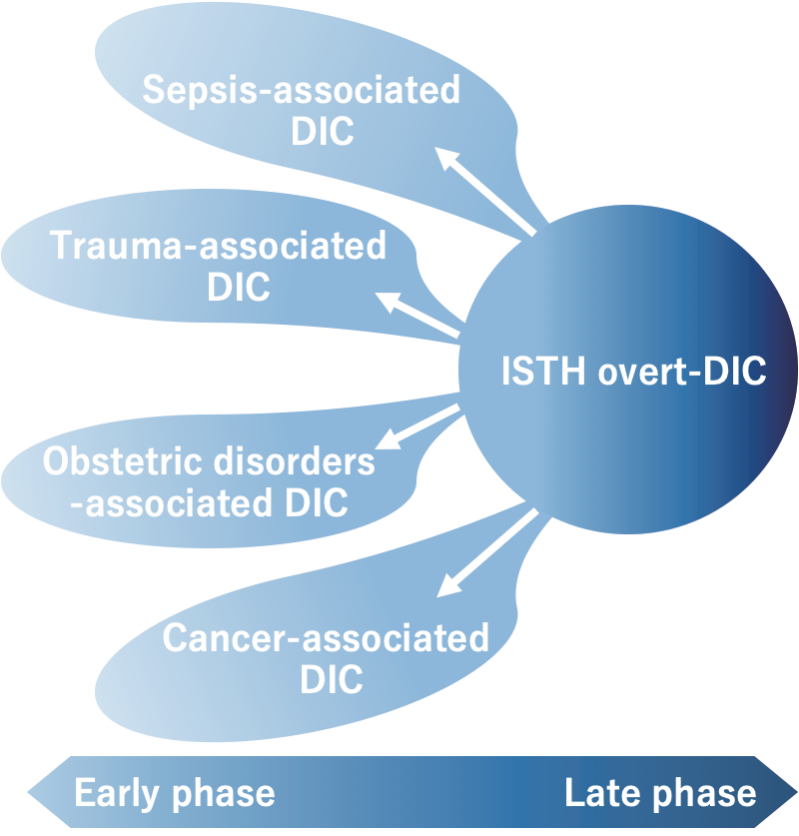
The trend toward the early and specific DIC diagnostic criteria Sepsis, trauma, obstetrical disorders, and cancers frequently serve as common underlying diseases for disseminated intravascular coagulation (DIC). While the International Society on Thrombosis and Haemostasis (ISTH) DIC criteria have been widely accepted as the standard, there is a growing need for simple and easily applicable DIC criteria that allow for early diagnosis specific to each underlying disease, given the advocated delay in diagnosis.

An answer to the above conundrum is considering DIC when i) the different laboratory tests are taken in conjunction; rather than in isolation which can overcome the issue of attributing the results to other causes, and ii) repeating the tests after a time interval that shows worsening test results, sometimes along with organ impairment. In this context, the development of the simple diagnostic criteria, i.e., sepsis-induced coagulopathy (SIC) criteria by the authors, has been extremely beneficial ^9)^ (Figure 1). Importance is given to the sequential organ failure assessment (SOFA) score AND the simple laboratory markers, platelet count, and prothrombin time in this multiply validated criteria for patients with sepsis ^10)^. Another big advantage of the SIC criteria is the ability to use it in low-resource settings since the components are easily performed. Other than sepsis, a pregnancy- specific score for diagnosis of DIC in obstetrics has also been validated in multiple studies ^11)^.

## Looking to the future

So far, endothelium and its biomarkers of injury have been overlooked in the clinical and diagnostic work-up of patients with DIC. The inclusion of the SOFA score in the SIC criteria is a significant step forward in this regard to include disease severity scores. However, despite several studies showing the crucial role of the vascular endothelium in the pathophysiology of DIC, the biomarkers included in these studies have not yet become mainstream and as such, cannot be widely recommended at the current stage for DIC diagnosis without prospective trials and validation studies. What can be done until then is to go back to the basics, i.e., using the ISTH definition and more usage of diagnostic criteria (e.g., SIC) among specialists who deal with the conditions that lead to DIC. Based on this consideration, this will allow earlier diagnosis of more patients with DIC at a stage where therapeutic measures can potentially reverse the uncontrolled intravascular coagulation activation and limit its dissemination.

## Funding

No funding was received.

## Author contributions

JT wrote, and TI, ES, and JHL reviewed and revised the manuscript. All authors read and approved the final manuscript.

## Conflicts of interest statement

The authors declare that they have not conflict of interest.
